# Prognostic factors of neuroblastoma in limited-resource settings

**DOI:** 10.1590/1984-0462/2025/43/2024200

**Published:** 2025-07-28

**Authors:** Roberta Gomes Ribeiro Gonçalves Pinto, Mecneide Mendes Lins, Kaline Maria Maciel de Oliveira Pereira, Ticiana Ester Mattos Pascoal Meira, Eduarda Coutinho Albuquerque Neiva Coêlho, Alice Rodrigues Barbosa de Moraes, Marina Lundgren de Melo Batista, Leticia Ribeiro Maciel Pereira, Maria Júlia Gonçalves de Mello

**Affiliations:** a Instituto de Medicina Integral Prof. Fernando Figueira – Recife (PE), Brazil.

**Keywords:** Neuroblastoma, Oncology, Prognosis, N-MYC proto-oncogene protein, L-Lactate dehydrogenase, Neuroblastoma, Oncologia, Prognostico, Proteína proto-oncogênica N-Myc

## Abstract

**Objective:**

This study aimed to analyze survival and risk factors for death in children with neuroblastoma that may contribute to a viable risk classification for low-income countries.

**Methods:**

A historic cohort involving patients under 19 years of age with neuroblastoma was followed at a reference center in Northeast Brazil between July 2005 and July 2020. Data on sociodemographic, were collected. The outcomes studied were recurrence, disease progression, and five-year mortality. Multivariate analysis of Cox proportional hazards for death was performed. Overall and eventfree survivals were evaluated using the Kaplan-Meier method and the comparison between the groups studied, by the log-rank test.

**Results:**

Patients (n=126) were predominantly female with a median age of 26.5 months. Most presented primary adrenal, tumor stage 4 according to the International Neuroblastoma Staging System, unfavorable histology, and median serum lactate dehydrogenase (LDH) levels of 640.5 U/L. LDH≥640.5 U/L (hazard ratio [HRa] 2.49; 95% confidence interval [CI] 1.57–3.95; p<0.001) and stage 4 (HRa 1.67; 95%CI 1.00–2.78; p=0.047) were identified as risk factors for death. The overall survival was 32.4%, showing distinct curves regarding LDH serum levels and staging (log-rank p<0.05); the event-free survival was 26.3%.

**Conclusions:**

Elevated LDH serum levels were a risk factor for five-year mortality, and can be utilized as a prognostic marker in resource-limited settings.

## RESUMO

**Objetivo:**

O objetivo deste estudo foi analisar sobrevida e fatores de risco para óbito em crianças com neuroblastoma que possam contribuir para uma classificação de risco viável para países de baixa renda.

**Métodos:**

Coorte histórica envolvendo menores de 19 anos com neuroblastoma acompanhados em um serviço de referência no nordeste do Brasil entre julho de 2005 e julho de 2020. Foram coletadas variáveis sociodemográficas, clínicas e laboratoriais, características tumorais e evolução clínica. Os desfechos estudados foram recidiva, progressão de doença e óbito em cinco anos. Foi realizada análise multivariada dos riscos proporcionais de Cox para o óbito. A sobrevida global e a livre de eventos foram avaliadas pelo método Kaplan-Meier e a comparação entre os grupos estudados, pelo teste de log-rank.

**Resultados:**

Os pacientes (126) eram predominantemente do sexo feminino com mediana de idade de 26,5 meses. A maioria apresentava tumor primário em adrenal, estágio 4 de acordo com o Sistema Internacional de Estadiamento do Neuroblastoma, histologia desfavorável e mediana dos níveis séricos da desidrogenase láctica (DHL) de 640,5 U/L. DHL≥640,5 U/L (HRa 2,49; intervalo de confiança [IC]95% 1,57–3,95; p<0,001) e estágio 4 (HRa 1,67; IC95% 1,00–2,78; p=0,047) foram identificados como fatores de risco para óbito. A sobrevida global foi de 32,4%, com curvas diferentes em relação à DHL e ao estágio (log-rank p<0,05); e a sobrevida livre de eventos foi de 26,3%.

**Conclusões:**

Os níveis séricos elevados de DHL foram fatores de risco para óbito em cinco anos e podem ser utilizados como um marcador prognóstico em ambientes com recursos limitados.

## INTRODUCTION

 Neuroblastoma (NB) is the most common extracranial solid tumor in childhood. Although NB presents elevated rates of spontaneous regression, high-risk cases have the lowest potential for cure among pediatric neoplasia.^1,2^

 Clinical heterogeneity is one of the main characteristics of NB. Age at diagnosis, disease stage, histological characteristics, and genetic variations (e.g., *MYCN gene* amplification, tumor cell ploidy, and segmental chromosomal alterations in 1p, 11q, and 17q) are the major prognostic factors.^3-7^ Additionally, somatic mutations in the anaplastic lymphoma kinase (*ALK*) gene, found in approximately 10% of sporadic cases, are associated with lower chances of cure.^8^ Serum levels of ferritin and lactate dehydrogenase (LDH) levels, although nonspecific, also correlate with prognosis.^3,9^

 Prognostic factors combined are used to classify patients into low-, intermediate-, and high-risk according to the International Neuroblastoma Risk Group (INRG). Patients classified as high-risk demonstrate low survival rates despite therapeutic advances. Conversely, low- and intermediate-risk patients, especially in high-income countries, have achieved excellent survival rates, especially after less aggressive therapies.^3^

 Understanding the clinical and biological factors is crucial for appropriate treatment.^1,10-12^ However, assessing genetic factors in low- and middle-income countries remains challenging.^13,14^ One study reported the correlation between gene amplification and MYCN protein expression evidenced by immunohistochemistry.^15^ Nonetheless, evidence suggests this protein may be overexpressed when the oncogene is not amplified, acting as an independent factor of poor prognostic.^16,17^ Therefore, evaluating MYCN protein expression may be a feasible alternative to assess the MYCN proto-oncogene, an important genetic biomarker.^15^ Thus, the present study aimed to analyze survival and risk factors for mortality in a cohort of individuals diagnosed with NB using clinical analysis and low-cost laboratory tests, contributing to minimum risk and feasible standardization of procedures for low- and middle-income countries. 

## METHOD

 This is a historic cohort study involving pediatric patients admitted to Instituto de Medicina Integral Prof. Fernando Figueira (IMIP) between July 1, 2005 and July 31, 2020, and followed up until July 2022. Eligibility criteria included patients under 19 years old diagnosed with NB. IMIP is a reference center for pediatric oncological treatment located in Northeastern Brazil. 

 Biopsy and histopathological examination, or analysis of infiltrated bone marrow and high levels of catecholamines and metabolites in serum or urine were used for diagnosis. Patients transferred to other services, with insufficient data, who underwent any treatment before admission to IMIP or the definitive diagnosis, were excluded. 

 Patients were identified through the institution’s database. Subsequently, the files were analyzed to verify the eligibility criteria and gather relevant information. Sociodemographic (age, gender, race, and geographical origin), clinical and laboratory variables (primary signs and symptoms, time between symptom onset and diagnosis, primary tumor site, metastases sites, staging, histological classification, serum levels of ferritin and LDH, MYCN protein expression, and gene amplification), and variables associated with treatment and patient outcomes were assessed. 

 The tumor staging followed the International Neuroblastoma Staging System (INSS) criteria.^4^ Available paraffin blocks were used for histopathological review, according to the International Neuroblastoma Pathology Committee (INPC) guidelines.^5^ A MYCN rat monoclonal antibody (Clone NCM II; catalog no. 16898100; Abcam, Cambridge, MA) was used in immunohistochemistry to assess MYCN protein expression. Nuclear staining at any degree (strong or weak) and quantity (diffuse or focal) was considered positive. MYCN protein expression was evaluated on paraffin blocks pre- or post-chemotherapy. An external laboratory performed fluorescent in situ hybridization to amplify the MYCN gene. 

 Treatment response was assessed based in part on the International Neuroblastoma Response Criteria (INRC).^18^ Complete remission was defined as the absence of disease at the primary and metastatic sites. Disease progression encompassed the emergence of any new lesion, an increase in any measurable lesion by more than 25%, and metastasis in the bone marrow. Stable disease included cases not meeting the criteria for complete remission or disease progression. 

 The investigated outcomes were recurrence, disease progression, and death. Recurrence was defined as the reappearance of NB after complete remission, confirmed via histopathology, or when clinical, laboratory, or radiological findings suggested a restart of specific treatment. Death was considered as the patient’s decease due to any cause. 

 The overall survival (OS) corresponded to the time between diagnosis and death or until the estimated censoring point, which was the last assessment within five years. The event-free survival (EFS) corresponded to the time from diagnosis to recurrence, disease progression, death, or censoring. 

 The data were entered into REDCap^®^ and analyzed using Stata 13^®^ software. For descriptive analysis (sociodemographic, clinical, laboratory, treatment-related, and patient evolution characteristics [recurrence, disease progression, and death]), a frequency distribution (absolute numbers and percentages) and measures of central tendency and dispersion were performed. Multivariate Cox proportional hazard analysis estimated the hazard ratio (HR) and identified potential predictors of death. In the initial multivariate model, variables with p< 0.25 in univariate analysis were included, and through backward elimination, variables with p< 0.05 remained in the final model. 

 For multivariate analysis, variables were categorized using the following cutoff points: age at diagnosis (<18 months and ≥18 months); gender; origin (Recife and metropolitan region, inland of Pernambuco, and other states); time between symptom onset and diagnosis (<1 month and ≥1 month); primary tumor location (adrenal and non-adrenal); staging (INSS 4 and non-INSS 4); histological classification according to INPC (favorable and unfavorable); MYCN protein expression (negative and positive); and LDH serum level (<median and ≥median). Five-year OS and EFS were evaluated using the Kaplan-Meier method and survival curve comparison through log-rank test. 

 This study was approved by the Institutional Review Board of IMIP (CAAE: 56270822.6.0000.5201). Patients signed the informed consent form and informed assent form when applicable. The signature was waived when contact was impossible or when the patient died. 

## RESULTS

 Out of 157 patients admitted over 15 years, 126 were diagnosed with NB, 1 with nodular ganglioneuroblastoma (GNB), 7 with mixed GNB, and 23 with ganglioneuroma. The 126 patients with NB were followed for approximately 16 years (median 23.1, interquartile range 11.2–59.5 months). 

Table 1 details epidemiological, clinical, and laboratory characteristics. Most patients were females (1.5:1 ratio), over 18 months old at diagnosis (65.1%), with two patients over 10 years old. 

**Table 1:** Epidemiological and clinical characteristics of 126 patients diagnosed with neuroblastoma from 2005 to 2020 at the Pediatric Oncology of Instituto de Medicina Integral Prof. Fernando Figueira.

Variables		Variables	
Age	months	INSS staging	n (%)
Extremes of age	0–183	1	4 (3.2)
Median (interquartile range)	26.5 (12–50)	2A, 2B	4 (3.2)
Mean±SD	34.8±30.7	3	36 (28.6)
		4	77 (61.1)
Sex	n (%)	4S	5 (3.9)
Male	51 (40.5)		
Female	75 (59.5)	Metastasis location*	(n=82) n (%)
		Bone Marrow	59 (46.8)
Race (n=51)	n (%)	Bone	56 (44.4)
White	21 (41.2)	Lymph nodes	28 (22.2)
Black	11 (21.6)	Liver	16 (12.7)
Brown	18 (35.3)	Skin	2 (1.6)
Indigenous	1 (1.9)	Others	7 (5.6)

Origin	n (%)	Histology – INPC (n=51)	n (%)
Recife	25 (19.8)	Favorable	20 (39.2)
Metropolitan area, excluding Recife	33 (26.2)	Unfavorable	31 (60.8)
Inland regions	61 (48.4)		
Other states	7 (5.6)	LDH	U/L
		Value extremes	161–7,962
Time from symptom onset to diagnosis	months	Median (interquartile range)	640.5 (393.5–1,235.5)
Extreme value	0–12	Mean±SD	1,163±13,016
Median (interquartile range)	1 (0–2)		
Mean ± SD	1.62±2.14	Ferritin (n=8)	ng/mL
		Value extremes	45–1,650
Primary tumor location	n (%)	Median (interquartile range)	544.5 (64.5–946.5)
Adrenal	66 (52.4)	Mean±SD	600.7±578.9
Retroperitoneum, except adrenal	36 (28.6)		
Thorax	18 (14.3)	MYCN protein (n=56)	n (%)
Pelvis	4 (3.2)	Negative	52 (92.9)
Cervical region	2 (1.5)	Positive	4 (7.1)

* The same patient may have metastasis in more than one site. INSS: International Neuroblastoma Staging System;^24^ SD: standard deviation; INPC: International Neuroblastoma Pathology Committee;^6^ LDH: lactate dehydrogenase.

 The most frequent initial signs and symptoms were fever (44.4%), increased abdominal volume (41.3%), abdominal pain (30.2%), and bone pain (20.6%). Three patients had Kinsbourne syndrome, and three were asymptomatic. The time between symptom onset and diagnosis ranged from 1–12 months (median 1, interquartile range 0–2 months). 

 The primary site was predominantly the adrenal (52.4%); 61.1% of patients were INSS stage 4, and 46.8% had bone marrow metastasis. LDH serum levels were quantified in 124 patients, ranging from 161–7,962 U/L, median of 640.5 U/L, which corresponds to approximately three times the normal reference value. Histological classification and MYCN protein expression were assessed in 51 and 56 patients, respectively. Of these, 60.8% exhibited unfavorable histology, and 92.9% did not express MYCN. *MYCN* oncogene status was evaluated in 15 patients before protein expression assessment. One patient exhibited gene amplification, and five exhibited gene amplification and MYCN protein expression; none showed discordant results. 

Table 2 summarizes the treatment response and outcomes. At the end of the chemotherapy, 40 patients achieved complete remission (31.7%), 9 (7.1%) had progression, and 51 (41.0%) remained stable, of which 28 (54.9%) subsequently died, and 19 did not show signs of progression until the end of follow-up. Among patients that achieved complete remission, 24 (60.0%) experienced recurrence. The main recurrence sites were bone (75.0%), primary site (50.0%), bone marrow (45.8%), liver (12.5%), central nervous system (8.3%), and lung (8.3%). Only 6 patients had isolated recurrence, 4 in the bone and 2 in the central nervous system. 

**Table 2:** Distribution of treatment variables of 126 patients with neuroblastoma according to disease progression at the Pediatric Oncology of Instituto de Medicina Integral Prof. Fernando Figueira from 2005 to 2020.

Variables related to treatment and evolution	Frequency		Evolution			
	n	(%)	Relapse/Progression n (%)		Death n (%)	
			n	(%)	n	(%)
Chemotherapy	122	(96.8)	63	(51.6)	82	(67.2)
Surgery	63	(50.0)	-	-	-	-
Complete resection	40	(31.7)	26	(65.0)	20	(50.0)
Partial resection	20	(15.9)	11	(55.0)	10	(50.0)
Biopsy (second look)	3	(2.4)	2	(66.7)	2	(66.7)
HSCT + cis-retinoic acid	7	(5.6)	-	-	-	-
In the first remission	5	(4.0)	3	(60.0)	1	(20.0)
In relapse	2	(1.6)	2	(100.0)	0	(0.0)
Radiotherapy						
Post-HSCT	2	(1.59)	1	(50.0)	0	(0.0)
At primary tumor in the absence of HSCT	11	(8.7)	7	(63.6)	5	(45.5)
Response after induction (chemotherapy±surgery)						
Complete remission	40	(31.7)	24	(60.0)	18	(45.0)
Stable disease	51	(40.5)	28	(54.9)	29	(56.9)
Disease progression	9	(7.1)	9	(100.0)	9	(100.0)
Death during induction	26	(20.6)	-	-	-	-
Causes of death (n=82)						
Infection	25	(30.5)	-	-	-	-
Disease progression	49	(59.8)	-	-	-	-
Other causes*	8	(9.8)	-	-	-	-

HSCT: hematopoietic stem cell transplantation.

* Other causes of death: respiratory failure due to Pepper syndrome or the presence of a posterior mediastinal mass.

 We identified 18 patients who completed treatment and remained with stable residual tumors for at least three years without progression. Clinical and pathological characteristics and therapy administered to those patients are detailed in Table 3. All patients underwent chemotherapy, either with or without radiotherapy, partial resection, or biopsy. The follow-up time ranged from 3 to 16 years. 

**Table 3:** Characteristics and evolution of the 18 patients after treatment completion showing stable disease and remaining without disease progression at the Pediatric Oncology of Instituto de Medicina Integral Prof. Fernando Figueira from 2005 to 2020.

Patient	Age (months)	Sex	INSS	LDH	INPC	MYCN (IHC)	Chemotherapy	RxT	Surgery	Surveillance (years)
1	87	F	3	214	-	-	A	-	Biopsy	11
4	22	F	3	1016	-	-	A, B, C	LT	Biopsy	14
11	3	F	4	1555	-	-	A, B	-	-	16
23	7	M	3	482	-	-	A, B	-	PR	14
38	26	F	3	291	Unf	N	A, B	-	PR	13
59	21	F	3	442	-	-	A, B, C	LT	Biopsy	12
75	9	M	3	321	-	-	A	-	Biopsy	10
78	74	F	3	790	-	N	A, B	LT	PR	11
82	14	F	3	324	Fav	-	A	-	Biopsy	10
96	12	F	4	533	-	-	A	-	Biopsy	9
98	10	F	4	296	-	-	A, B	-	PR	9
104	11	F	4	654	-	-	A, B	-	-	7
105	12	F	4	359	-	-	A, B	-	-	7
125	5	F	4	679	-	-	A, B	-	-	6
127	14	F	4	497	-	-	A, B, C	LT	-	6
149	10	F	3	170	Fav	N	D	-	PR	3
153	13	F	4	491	Fav	N	D	-	Biopsy	3
154	11	M	3	455	Fav	N	D	-	PR	3

INSS: International Neuroblastoma Staging System;^24^ LDH: lactate dehydrogenase; INPC: International Neuroblastoma Pathology Committee;^6^ IHC: immunohistochemistry; RxT: radiotherapy; F: female; M: male; Unf: unfavorable; Fav: favorable; PR: partial resection; LT: tumor bed. A: NB84 – SJCRH;^29^ B: NB-91 SJCRH;^30^ C: four cycles of carboplatin (560 mg/m^2^) on day one, ifosfamide+etoposide+mesna on days two, three, and four; D: cycles one, two, five, and six – cyclophosphamide (250 mg/m^2^/day)+topotecan (0.75 mg/m^2^/day) days one to five. Cycles three, four, seven, and eight – cyclophosphamide (200 mg/m^2^/day) days one to seven+doxorubicin (45 mg/m^2^/day) day eight+vincristine (1.5 mg/m^2^) days 14 and 28+carboplatin (200 mg/m^2^/day) + etoposide (300 mg/m^2^/day) days 20, 21, and 22.

 During five-year follow-up, 82 (65.1%) patients died. The primary causes of death were disease progression (59.8%) and infection (30.5%). Respiratory failure secondary to Pepper syndrome or mediastinal mass was reported in 9.7% of cases. Deaths due to surgical complications did not occur. Twentysix (31.7%) patients died during the induction therapy phase due to complications and treatment-related toxicity, predominantly affecting those under 18 months of age with favorable characteristics (61.5%). 

Table 4 presents risk factors for five-year mortality. In the initial multivariate model, age group, gender, origin, primary tumor location, and INSS 4 were included compared to other stages and LDH serum levels. Age group was not a risk factor in univariate or multivariate analyses. However, for biological plausibility, multivariate results were adjusted for age, with LDH serum levels above 640.5 U/L (HR 2.49; 95%CI 1.57–3.95; p<0.001) and INSS 4 remaining in the final model (HR 1.67; 95%CI 1.00–2.78; p=0.047). 

**Table 4:** Characteristics of 126 patients with neuroblastoma and bivariate analysis according to the occurrence of death at the Pediatric Oncology of Instituto de Medicina Integral Prof. Fernando Figueira from 2005 to 2020.

	All patients		Survivors		Deaths		HR	95%CI	p-value
	n	(%)	n	(%)	n	(%)			
Age group (months)									
<18	44	(34.9)	23	(52.3)	21	(47.7)	1.49	0.91–2.45	0.116
≥18	82	(65.1)	21	(25.6)	61	(74.4)	1.00		
Sex									
Male	51	(40.5)	12	(23.5)	39	(76.5)	1.53	0.99–2.37	0.053
Female	75	(59.5)	32	(42.7)	43	(57.3)	1.00		
Origin									
Recife and metropolitan area	58	(46.0)	27	(46.5)	31	(53.5)	1.00	1.14–2.79	0.012
Inland regions and other states	68	(54.0)	17	(25.0)	51	(75.0)	1.78		
Time from symptom onset to diagnosis (month)									
<one	48	(38.1)	14	(29.2)	34	(70.8)	1.25	0.80–1.94	0.322
≥one	78	(61.9)	30	(38.5)	48	(61.5)	1.00		
Primary tumor									
Adrenal	66	(52.4)	18	(27.3)	48	(72.7)	1.48	0.95–2.30	0.080
Non-adrenal	60	(47.6)	26	(43.3)	34	(56.7)	1.00		
Staging (INSS)									
1, 2A, 2B, 3, 4S	49	(38.9)	27	(55.1)	22	(44.9)	1.00	1.28–3.41	0.003
4	77	(61.1)	17	(22.1)	60	(77.9)	2.08		
Histology (INPC) (n=51)									
Favorable	20	(39.2)	11	(55.0)	9	(45.0)	1.00	0.85–3.97	0.119
Unfavorable	31	(60.8)	7	(22.6)	24	(77.4)	1.84		
LDH U/L (n=124)									
<640.5	62	(50.0)	33	(53.2)	29	(46.8)	1.00	1.67–4.17	<0.001
≥640.5	62	(50.0)	9	(14.5)	53	(85.5)	2.64		
MYCN protein (n=56)									
Negative	52	(92.9)	21	(40.4)	31	(59.6)	1.00	0.75–6.21	0.153
Positive	4	(7.1)	0	(0.0)	4	(100)	2.16		

HR: Hazard ratio; CI: confidence interval; INSS: International Neuroblastoma Staging System;^26^ INPC: International Neuroblastoma Pathology Committee;^7^ LDH: lactate dehydrogenase; 640.5 U/L: LDH median.

**Figure 1:**
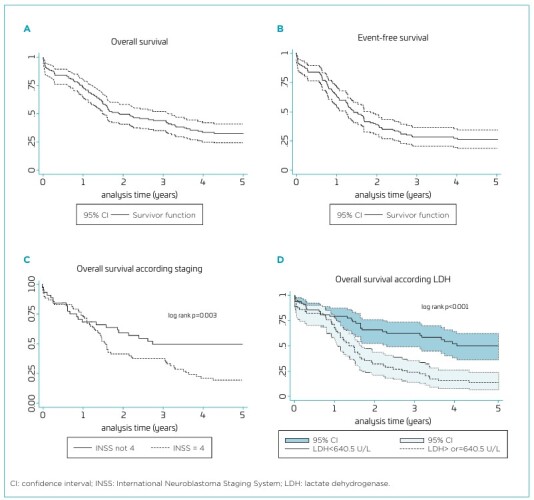
Cohort of 126 patients with neuroblastoma followed at the Pediatric Oncology of Instituto de Medicina Integral Prof. Fernando Figueira from 2005 to 2020. A. Overall survival; B. Event-free survival; C. Overall survival according to staging; D. Overall survival according to lactate dehydrogenase.

 The five-year OS was 32.4% (95%CI 24.1–40.9%), and EFS was 26.3% (95%CI 18.8–34.4%). In OS curve analysis, LDH serum levels (35.5%; 95%CI 23.9–47.3% vs. 12.9%; 95%CI 6.022.5%; p<0.001) and staging (53.9%; 95%CI 38.8–66.8% vs. 19.3%; 95%CI 11.1–29.4%; p=0.003) differed significantly (Figure 1). Analysis of OS by age group revealed no statistically significant differences. Similarly, no significant findings were observed for patients with INSS 3 across all age groups, as well as for those with INSS 4 under 18 months, based on LDH categorization. Detailed results for the latter are not presented in figures. 

## DISCUSSION

 In this study, the mean age of the patients with NB was approximately 35 months; most were females and had metastasis at diagnosis. Patients had OS and EFS less than 30%, and those with LDH serum levels ≥640.5 U/L and INSS 4 presented a higher risk of five-year mortality. 

 The incidence and mean age at diagnosis varied significantly.^14,19^ Previous reports have shown an association of these variables with socioeconomic conditions and the implementation of screening programs.^14,19,20^ In high-income countries with access to health care services and imaging tests and countries with screening programs in place, the diagnoses increased in patients under 12 months with tumors likely to regress spontaneously. In those contexts, the mean age was 18 months, of which 40% were under one year.^20^ In resource-limited countries, the mean age at diagnosis was over 18 months, with most presenting metastasis.^14,19^

 Corroborating the literature, many patients had primary tumors in the adrenal with nonspecific symptoms, which might have delayed the diagnosis.^21,22^ The three patients with Kinsbourne syndrome exhibited favorable biological characteristics and good clinical outcomes.^3,12^

 In the present study, most cases had unfavorable histology and higher LDH serum levels, differing from the results of a large INRG cohort, possibly due to the higher number of patients older than 18 months and its association with adverse biological features.^3,6^ The positivity rate to MYCN gene and protein expression analyzed in a subgroup was lower than reported in the literature.^3,12^ Patients were under 18 months old with INSS non-4, which is a potential selection bias. Moreover, the tumor sample was obtained after chemotherapy in half of the patients with INSS 4. Due to the histological differentiation, the sample characteristics probably influenced MYCN protein expression.^17^ The proportion of patients with gene amplification and MYCN protein expression was similar. 

 After treatment, the clinical evolution of patients revealed a higher percentage of stable disease. Among patients with stable disease, 18 were followed up for at least three years without signs of progression until the end of data collection. Despite the presence of unresectable residual tumors, favorable prognoses were observed in all patients. This is a retrospective study, not all biological information required for risk assessment were available; however, based on clinical evolution, these patients were possibly within the intermediate-risk group.^11,23-25^

 The findings from the multivariate analysis align with previously reported data in the INRG cohort, indicating lower EFS for patients with INSS 4 compared to those with INSS non-4 and with LDH serum levels higher than 587 U/L.^3^ In another study, patients with INSS 3 and high LDH serum levels (>580 U/L) experienced low EFS.^24^ LDH can be valuable as a prognostic biomarker even within the high-risk group, particularly in low-income countries, where access to molecular evaluation is limited and often unavailable.^9^ In the management guidelines for NB recommended by the Pediatric Oncology in Developing Countries committee, LDH was considered one of the markers to differentiate patients with favorable and unfavorable risks.^13^

 In previous studies, age over 18 months and unfavorable histological classification were associated with a higher risk of death, which was not observed in the present study.^3,4,6^ This difference could be justified by the small number of patients undergoing histological classification and the high death rate during chemotherapy in patients under 18 months. In the first years of this cohort, patients were classified according to tumor staging and age; patients under one year were considered lower risk. Thus, patients between 12 and 18 months received more intensive treatments, resulting in higher toxicity. 

 In this study, the median time between the first signs and symptoms and diagnosis was one month, and this interval was not associated with survival. Although early diagnosis is usually important in childhood cancer, studies in children with NB have not consistently shown this association. Screening programs implemented in North America, Germany, and Japan led to an "increase in incidence", mainly among patients under one year old, who would likely regress spontaneously and would not be diagnosed without screening. However, detecting advanced forms in all age groups remained the same after the implementation of these screening programs and did not impact survival.^12^

 Investigating the *MYCN* oncogene is fundamental for risk stratification. However, this test is expensive and less accessible in low- and middle-income countries.^13,15^ To make *MYCN* assessment available in these regions, St. Jude Children’s Research Hospital (Memphis, USA) conducted a study, evaluating the correlation between MYCN protein expression by immunohistochemistry and gene amplification by fluorescent in situ hybridization. Most patients with amplified *MYCN* strongly expressed the protein.^15^ Nevertheless, MYCN protein overexpression can occur without oncogene amplification, and in these cases, the prognostic value remains controversial.^26^ Some studies have attempted to demonstrate the prognostic value of MYCN protein expression in NB.^26,27^ In a study involving 69 patients with INSS 3 and 4 with non-amplified genes, the level of MYCN expression was not predictive of outcomes.^26^ Conversely, more recent studies from the Children’s Oncology Group and those conducted in China showed conflicting results. These works indicated that patients with protein overexpression had an unfavorable outcome independent of gene amplification.^16,17,27^

 After these publications, the pediatric oncology service at IMIP determined this marker through immunohistochemistry as part of the protocol. As a retrospective study, only the samples of 56 patients were available and evaluated. The results indicated that MYCN protein expression was not a poor prognostic factor. This may have occurred due to the sample size, heterogeneity of the group, and the fact that some patients with INSS 4 were assessed after chemotherapy, which may have led to negative results.^17,27^ Nevertheless, all patients expressing MYCN protein showed other characteristics and unfavorable outcomes. Thus, future studies with a larger sample should further evaluate the prognostic value of MYCN protein expression and its association with gene amplification. 

 The OS of patients with NB varies according to the risk, being over 90% in low-risk and around 50% in high-risk patients.^10,11,28^ In this study, OS and EFS rates were lower than those found in high-income countries. This finding may be related to the higher percentage of patients under 18 months with advanced-stage disease and deaths related to treatment.^18^

 A significant difference in LDH serum levels and staging was reported in the analysis of OS in different groups.^9^ The OS did not differ when assessing patients with INSS 3 of all ages and with INSS 4 under 18 months based on LDH serum levels. However, the small sample size influenced this evaluation. Therefore, further studies are necessary to define the prognostic value of LDH. 

 This cohort followed a substantial number of patients for years; however, it has limitations as a retrospective study from a single center. Also, the lack of information regarding histological classification and MYCN protein expression may have influenced some results. Thus, multicenter prospective studies with standardized assessment may contribute to a better understanding of disease prognosis. 

 Therefore, NB is a highly heterogeneous neoplasia, posing a challenge for pediatric oncology, especially in low- and middle-income countries where the limited capacity to adequately classify risk decreases the chances of cure.^15^ In these settings, LDH serum levels are demonstrated to be a risk factor for five-year mortality, and can be utilized as a prognostic marker in resource-limited settings.^9^

## Data Availability

The database that originated the article is available with the corresponding author.
